# Cognitive strategies revealed by clustering eye movement transitions

**DOI:** 10.16910/jemr.13.1.1

**Published:** 2020-02-26

**Authors:** Šimon Kucharský, Ingmar Visser, Gabriela-Olivia Truțescu, Paulo G. Laurence, Martina Zaharieva, Maartje E. J. Raijmakers

**Affiliations:** University of Amsterdam, the Netherlands; Leiden University, the Netherlands; Mackenzie Presbyterian University, São Paulo, Brazil; Vrije Universiteit, Amsterdam, the Netherlands

**Keywords:** Cognitive strategies, eye tracking, unsupervised clustering, latent groups, scanpaths

## Abstract

In cognitive tasks, solvers can adopt different strategies to process information which may lead to different response behavior. These strategies might elicit different eye movement patterns which can thus provide substantial information about the strategy a person uses. However, these strategies are usually hidden and need to be inferred from the data. After an overview of existing techniques which use eye movement data for the identification of latent cognitive strategies, we present a relatively easy to apply unsuper-vised method to cluster eye movement recordings to detect groups of different solution processes that are applied in solving the task. We test the method's performance using simulations and demonstrate its use on two examples of empirical data. Our analyses are in line with presence of different solving strategies in a Mastermind game, and suggest new insights to strategic patterns in solving Progressive matrices tasks.

## Introduction

Traditionally, response behavior such as accuracy and more recently response time are typically used to make inferences about participants' cognitive states, processes, or abilities to solve cognitive tasks [1, 2]. Eye movements are a valuable source of information which extends our ability to make this kind of inference [e.g., 3].

However, analyzing eye-tracking data is a challenging problem especially when cognitive strategies are to be inferred from the locations at which participants look and the order in which they look at them. Analyzing the information about the spatial and temporal dimensions of eye movements is commonly referred to as scanpath analysis, where the term scanpath concerns the spatio-temporal sequence of fixations and saccades, a term coined by Noton and Stark [4].

Pioneering work of Yarbus [5] showed [among other discoveries, 6] that giving different instructions to observers changes their gaze behavior. This inspired the eye-tracking research community to devote its attention towards the so called inverse Yarbus problem [7, 8]: in this area of research, the question is whether it is possible to infer a task or a strategy from eye movement patterns rather than whether a task or strategy invokes different gaze behavior. Most of the applications to investigate the inverse Yarbus problem deal with situations where we know what task or strategy a participant uses, e.g., using an experimental manipulation or recruitment based on diagnosis or a cognitive development stage. This enables researchers to use supervised techniques to show that some form of eye-tracking data representation can be used to describe the strategy of the observed groups. The representations range from similarity measures based on string edit and sequence methods [9, 10, 11, 12], classifying raw eye tracking statistics [7, 13, 14, 15, 16], Markov models [1, 17] or hidden Markov models [8, 18, 19, 20]. For a review of different approaches to predict a task from eye movements see Boisvert and Bruce [14]. However, the question of how to infer a task or a strategy is a topical issue especially when it is unobserved (i.e., latent) and has to be inferred from the eye movements alone (i.e., a latent inverse Yarbus problem). This is precisely the issue of the current study.

### Discovering latent groups

Latent groups are of interest whenever there is a reasonable expectation that the observers might use a set of qualitatively different approaches to the task, and these differences would manifest through their gaze behavior, but it is unknown which observer uses which approach on which stimuli, other than what can be inferred from the eye movements themselves. This distinguishes the latent group problem from the prediction problem. In the prediction of a task, one has the information about the groups of observers which are supposed to qualitatively differ in the eye movement patterns and needs to learn which features of eye movements discriminate between these groups. In the latent group problem, one has to learn about the presence or absence of qualitatively distinct groups, and identify the features of the eye movements that are characteristic of these groups. The discussion of latent groups manifesting through eye movements appear in the context of cognitive tasks [10, 21, 22, 23, 24], decision making [25, 26], visual search tasks [27], face recognition and exploration [28, 29, 30, 31], and various other topics [20, 33, 34].

In the context of cognitive tasks, the detection of qualitatively distinct groups of eye movements can be especially informative, because the groups might be related to a cognitive strategy a person uses to solve the problem at hand [35, 36]. Using the eye-tracking patterns to identify these strategies can bring additional insights as to how people solve these problems and can thus complement more conventional analyses of response behavior [37, 38, 39].

Detecting latent groups from eye movements can be viewed similarly as detecting latent groups from response behavior [37, 38], with the only difference being the type of data that are used as an input. Generally, the goal of detecting strategies can be achieved by unsupervised clustering methods or mixture modeling of the eye movement data. Unsupervised methods for clustering similar eye movement patters has already been used in context of face recognition [clustering hidden Markov models, e.g. 28, 29, 30], reading [latent profile modelling based on scanpath similarity measure, 12], free viewing [hierarchical clustering based on similarity measure, 33], visual search (manual classification, 27), or usability testing [40, 41], among others.

In the context of cognitive tasks hypotheses about latent solving strategies are currently not always tested using latent group analyses. In many cases, based on theoretical expectations on how the latent strategies should manifest, researchers first define aggregate statistics from the eye movements (e.g., number of transitions, frequency of transitions between different areas of interest, etc.). Then they relate them to performance, thereby showing that different strategies result in different eye movement statistics that are subsequently correlated with performance in the task at hand [21, 22, 42, 43], although an alternative approach has been proposed to model the eye-tracking data [Successor Representation Scanpath Analysis (SRSA), 23, 24]. In short, SRSA builds successor representation matrices which contain information about the higher order transition dependencies in the data, which are then reduced in smaller number of dimensions and used as predictors of performance in the task. By adjusting parameters that control the specification of these matrices, the methods searches for a solution which maximized the prediction of the task performance [i.e., a semi-supervised approach whereby task performance substitutes an indicator of the strategy, 23, 24]. It is often the case that the relationship between the latent strategy and the task performance (or other variable) is itself an empirical question. In this situation, conducting an unsupervised latent group analysis first will enable us to separate two questions from each other – first, whether we can detect qualitatively different eye movement patterns, and second, whether these patterns relate to performance (or other variables of interest). Crucially, this approach allows discovering groups that are not necessarily related to performance, and thus provides an opportunity to assess the latter question empirically. This distinction is important when the sole predictive performance is not of such an importance compared to assessing theories about qualitatively different cognitive processes, and to explain, rather than predict, individual differences [for in depth discussion of the trade-off between prediction and explanation, see 44].

### Eye movement representation

To conduct a latent group analysis, a choice needs to be made how to represent the eye movements data (in terms of its spatial and temporal features) to serve the purpose of finding the latent groups in the specific context. The need to choose between different representations arises due to the fact that the raw eye-tracking data are, in their totality, too complicated (and perhaps noisy) to provide meaningful insights into the phenomenon under investigation. Thus, researchers usually need to define which features of the data are meaningful or discriminatory for the specific application and model them as such. For example, many authors emphasize individual differences in the processing of facial features, resulting in a distinction between holistic and analytic strategies in face recognition [17, 28, 29, 30]. Thus, hidden Markov models are suitable for this purpose as they allow to identify the important parts of the stimulus in a bottom-up manner. Furthermore, the transition patterns of the hidden Markov model between the facial features enables to discriminate between left-eye biased and right-eye biased analytic patterns. Based on a careful consideration of the specifics of eye movements in reading, von der Malsburg and Vasishth [12] use their own similarity measure which does not require discretization of the stimulus into regions of interest and can take into account the fixation duration, which is important in the context of syntactic analysis of sentences. Another approach [33] relies on string edit distances [45, 46, 47] to cluster sequences based on similarities between pairs of eye movement recordings.

In case the stimuli can be unambiguously divided into distinct meaningful areas of interest and the number, shape and position of these areas is assumed to be constant between the latent groups [as is the case in many cognitive tasks, e.g., 21, 26, 48], a promising candidate for such representation is a transition matrix between pre-defined areas of interest, in which we quantify the probability of the next fixation on any area of interest conditionally on the position of the current fixation. Constructing or fitting transition matrices is relatively well established in the eye-tracking literature, either as descriptive statistic of the transition patterns [49, 50, 51] or as an integral set of parameters specifying (hidden) Markov models [see 18, 52, and references therein]. Compared to the hidden Markov models, constructing transition matrices from the fixated areas of interest significantly reduces the complexity of the analysis at the expense of binning fixations into pre-defined areas of interest instead of treating them as hidden states that need to be estimated from the data. This essentially simplifies the problem into a representation of categorical time-series [53], without the need for a complicated evaluation of the likelihood of each eye movement sequence as a whole as is the case in hidden Markov models. It is important to note that if the areas of interest cannot be defined in advance (e.g., because their position is itself a topic of empirical investigation), the data are too noisy relative to the sizes of the areas of interest, or if there are too many borderline fixations, this simplification may not be justified and other, more complex, approaches (e.g., hidden Markov Models) may be necessary.

Despite the fact that by using only the first-order transition matrices one potentially ignores informative features of the eye movements data [12, 23], they can still provide rich information about the transition patterns between the areas of interest, patterns which in many cases should be different between solution strategies that participants apply in cognitive tasks. Using transition matrices should be, in some cases (as we show later), sufficient to detect latent groups, while providing rich description of the characteristic features of the transition patterns that define these groups.

### Goals & outline

Following the arguments in the previous sections, we believe that a method for detecting latent groups from eye movement data would be informative to investigate the existence of different solution strategies in cognitive tasks, and eventually also their relation to task performance. Such a method should generally meet the following desiderata. First, the eye movement patterns should be analysed (summarised) such that the features of the hypothetical strategies can be detected. Second, the method should be unsupervised to allow detecting latent groups, even if they do not relate to external variables. Third, it should be possible to use some selection method for the number of such groups. Fourth, the classification of an eye movement pattern into a latent group should be possible on an individual item basis to allow the possibility that participants switch between strategies during the task (for example, due to learning).

This article demonstrates the use of transition matrices as a representation of eye movements in order to detect latent groups of similar eye movement transition patterns. Specifically, we use a relatively easy to apply unsupervised method to discover latent groups, and present ways in which the classifications can be used in further analyses.

The structure of the article is as follows. In the next section, we provide details about how to construct transition matrices, and present the method we use for their clustering. Next, using simulations, we show that this method is able to retrieve the groups corresponding to strategies for solving a Mastermind Game and present an application of the method to real data. Then, we apply the method to a data set of the Wiener Matrizen Test [a test very similar to the Raven’s Progressive Matrices; 43]. We conclude with discussion of our findings, as well as with the limitations, alternatives, and extensions to our approach.

## Clustering transition matrices

Our goal is to introduce a method that can be used for unsupervised clustering of eye movement sequences. Our approach is the following. First, we process the fixation coordinates into pre-specified areas of interest (AOIs). Such approach is typical in eye-tracking literature, at least in tasks with clearly distinct meaningful parts of the stimulus, although there is some discussion on how to optimally choose and delineate AOIs [e.g., 54].

From each individual sequence of the fixated AOIs, we create the transition probability matrix, where each row corresponds to a "sender" AOI, and each column to the "receiver" AOI. Each row of the matrix is computed by counting the number of transitions from the sender AOIs to all other AOIs and dividing the row by the sum of the total transitions from that AOI. The entries of the *d* × *d* transition probability matrix **M** can be interpreted as follows: Given that a fixation is on the *i* ^th^ AOI, the probability that the next fixation is on the *j* ^th^ AOI is equal to **M**_*ij*_.

All transition matrices are then reshaped into vectors of length *d* ^2^ and stored in a data matrix where the rows correspond to the individual matrices (i.e., representations of the individual AOI sequences), and columns correspond to the cells in the transition probability matrices. The resulting data matrix is then subjected to the standard *k*-means clustering algorithm [55].

As an unsupervised method, *k*-means provides us with an opportunity to find distinct groups of eye movement patterns, patterns which differ in their transition matrices, Scree plots can be used to diagnose solutions for different numbers of clusters. Furthermore, each cluster is assigned a mean transition matrix which identifies the characteristic features of the transition patterns in each group, which can be used to interpret the groups and assign a label according to hypothesized cognitive processing. We use standard *k*-means based on minimizing the within-cluster sum of squared Euclidean distances from the centroids to east of interpretation of the cluster centroids. Relative Euclidean distances of individual matrices to the cluster centers can be used to assess the representativeness of each eye movement recording of that particular group. The cluster assignment indicators can be used for further analysis, for example examining the relationship of the clusters to performance. The demonstration of our approach follows in the next two examples and a simulation study.

### Application: Deductive Mastermind

Here, we present an example of detecting cognitive strategies in a Deductive Mastermind Game (DMM). In the DMM, the player is supposed to deduct a sequence of flowers based on multiple "conjectures" composed of a sequence of flowers and their corresponding feedback presented as a collection of colors next to the conjecture. The green feedback means that a flower in the conjecture belongs to the solution, red feedback means that a flower does not belong to the solution, and orange feedback means that a flower belongs to the correct solution but is on a wrong place in the sequence [39].

The DMM was implemented as a part of web-based math and logic training system in primary schools called Math Garden (Rekentuin.nl or MathsGarden.com). Gierasimczuk et al. [39] analyzed data collected with Math Garden and revealed that the player ratings and the item difficulty have a tri- and bi-modal distributions, respectively. Logical analysis of the game showed that the items can be solved using different strategies, ones that vary in the number of steps a player needs to deduct the correct solution. One possible explanation for the multimodality of the player ratings might be that the population of players is a mixture of people using different strategies, strategies which relate to the efficiency in solving the game.

**Figure 1.**
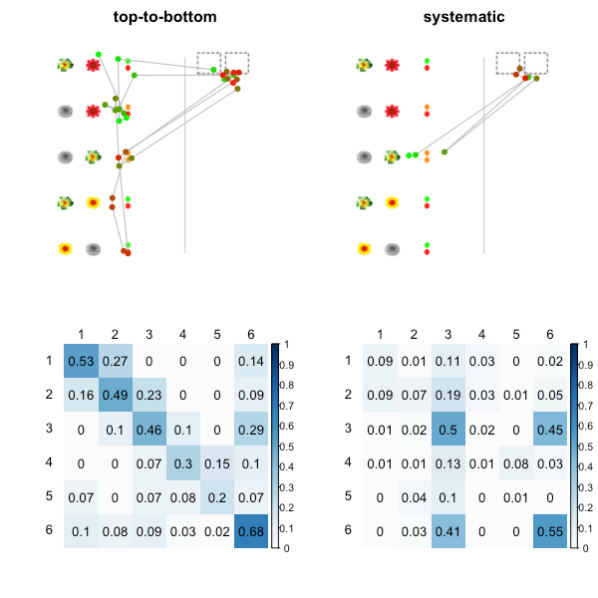
Synthetic data of two strategies in one of the selected Mastermind games. The left panel shows the top-to-bottom strategy, and the right panel shows the systematic strategy of selective processing. The top panel shows examples of the scanpaths, where dots correspond to fixations (the color gradually changes from bright green to dark red based on the order of the fixations) and lines connect the successive fixations (i.e., saccades). Bottom panel shows the transition matrices of the simulated strategies over 1,000 simulations.

The logical analysis predicts at least two strategies to occur during solving the items. The first strategy is characterized by scanning the feedback in the order in which it is presented (i.e., from top to bottom) – this prediction relies on the assumption that it is a natural (i.e., learned) way of processing information before internalizing the differences in information value that different feedback holds. The prediction of the second strategy, in contrast, relies on the fact that each row of the stimuli can have different information value. Thus, this strategy would be characterized by selectively scanning the feedback starting from the conjectures which contain the most information and proceeding to those which complement it. Figure 1 shows one of the items with superimposed eye-tracking patterns under the two strategies. Notice that the first order transition matrix differs between the strategies. Thus, it should be possible to discriminate between them using only the first order transition patterns.

### Methods

We use subset of the data that was collected with adults outside the educational system as part of a larger project [48]. The data are available at https://osf.io/he43s/. Twenty-six university students with normal or corrected-to-normal vision participated in the study. Two participants were excluded from the analysis due to missing data in the eye-tracking measurements. The study comprised of one learning block (13 items) and two test blocks (16 items each) in randomized order of the items within each block. The 2-pin items were constructed as an adapted version of the DMM task suitable for eye-tracking by adjusting the layout of the displayed conjectures [see Appendix in 48]. The items in the learning phase were designed such that they are easily solvable regardless of the scanning strategy; items with all combinations of feedback were presented to give participants the opportunity to establish the difference between feedback types. For a concise presentation of the current method, we further analyse only four items (two from each test block) containing orange-orange feedback at the third row. These items can be solved by focusing only on the third row, as the orange-orange feedback informs to swap the positions of the two presented flowers, see Figure 1.

The eye movements were recorded using EyeLink-1000 eye tracker with 500 Hz sample rate (SR Research Ltd., Ontario, Canada). Participants were seated at a desk with a chin rest about 55 cm in from of a 17-inch computer monitor, subtending an approximate 27 × 24 visual angle. Before the data collection, a five-point calibration was used, which was repeated until the recorder point of gaze reached the best possible quality.

The raw data were parsed into fixations and saccades using Gazepath algorithm [56]. The identified fixations were classified into sic rectangular areas of interest, one each for the row of the task in the left panel, and the sixth belonging to the response area on the right panel (see Figure 1). Thus, "scanpath" is in our case operationalized as a series of fixated AOIs. By doing so we artificially segregate the items into meaningful chunks of information: the units of information are the pairs of flowers and associated feedback, which corresponds to the definition of the conjectures in the logical analysis of the Mastermind game [39]. This approach imposes relatively strong assumptions on the semantic connotation (i.e., the structure of the task as interpreted by individual participants) and could be prone to measurement noise (in applications where the AOIs are relatively small or close to each other); we nevertheless consider this a sensible approach in the current task as the rows of the stimuli were designed to be visually well separated and correspond to the semantic denotation of the task, which had been communicated through the experimenter's instructions and demonstrated during the learning block.

*Transition matrix eye movement analysis.* Before clustering the data, we conducted a simulation study in order to investigate the method's performance. We previously analyzed all scanpaths on the four selected items for which classification into the top-to-bottom and systematic strategy was possible by visual inspection. We wrote a simulation function which mimics the two strategies (top-to-bottom and systematic, see Figure 1). The simulated patterns were matched with real data with respect to several criteria (see https://osf.io/82.fau/ and https://osf.io/ bz3ny/). This enabled us to simulated an arbitrary number of participants using one or the other strategy with some variability in the patterns within the strategies (associated R code at https://osf.io/jxwrk/).

We tested the method's performance with respect to two varying features of the simulated data. For each of the features, we selected three values, resulting in a 3 by 3 simulation design. The features we varied and their values were the following:

Sample size: *n* (20, 60, 100). We varied the total number of the participants in the simulated studies.

Proportion of strategies: *p* (0.25, 0.5, 0.75). We varied the proportion of participants using one or another strategy. The value of *p* corresponds to the proportion of participants using the top-to-bottom strategy. This number was treated as a sample proportion (not population proportion) and thus there was no sampling variance between the simulations using the same value.

We simulated 600 data sets per each combination of parameters (totaling 3 × 3 × 600 = 5,400 simulated studies). In each simulation, each participant solved only one item. This allowed us to inspect the robustness of the method even for item-wise analysis (i.e., with relatively sparse data).

For each data set, the procedure was follows. Each individual sequence of AOIs was converted to a 6 × 6 transition matrix. The individual matrices were reshaped into a vector of length 6 × 6 = 36 and stored into a *n* × 36 matrix. The *k*-means clustering was applied to this matrix with solutions from 1 to 10 clusters to inspect whether the scree plot identifies the correct number of clusters (2).

Next, we assumed that the correct number of groups was selected and investigated the classification accuracy of the two-cluster solution. We also investigated the stability and accuracy of the estimated cluster centers. To do this, we had to resolve an issue of label switching. In each simulation we created a 2 × 2 confusion matrix of the true group membership against the estimated labels given by the *k*-means. If the sum of the diagonal entries in this matrix was greater than the sum of the off-diagonal entries of the matrix, we kept the labels as they are. If this was not the case, the cluster indicators from *k*-means were relabeled.

After the simulations were conducted, we applied clustering of transition matrices to real data. The subset of the whole data consists of 24 × 4 = 96 eye movement sequences for cluster analysis. For each sequence, we calculated the 6 × 6 transition probability matrix and reshaped it into a vector of length 36. The resulting 96 × 36 matrix was subjected to the *k*-means clustering.

### Simulation results

*Extracting the correct number of strategies.* A rule of thumb for selecting the number of clusters is to inspect a scree plot to see at which point the amount of unexplained variance by the clusters stops decreasing rapidly. Given the subjective nature of this procedure, we cannot report exact number of the cases where the scree plot would identify the true number of latent groups (2) correctly. However, from a qualitative inspection of the scree plots, we saw that the classic "elbow" shape emerges mostly when 1) sample size is large, and 2) when the sized of groups are even. Decreasing the sample size results in mostly uninformative scree plots (i.e., the scree plot decreases gradually).

*Classification accuracy.* For all simulations, we inspected how accurate is the participant assignment using the solutions with two latent groups.

Table 1 shows the median and interquartile range of the assignment accuracy for all combinations of n and p. Overall, the classification accuracy is high, in most scenarios higher than 90 %. The total accuracy is the highest when the two strategies are equally represented in the sample, and slightly increases with sample size. The accuracy of correctly classifying top-to-bottom pattern is slightly lower than the accuracy of classifying the systematic pattern, which might be due to the fact that the top-to-bottom pattern is more variable, and can also contain characteristic features of the systematic pattern (namely, transitions from the third row to the response).

**Table 1:** Median and interquartile range of classification accuracy based on the k-means clustering of transition matrices.

p	n	Total	Systematic	Top-to-bottom
0.25	20	0.90 (0.75, 0.95)	0.93 (0.80, 1.00)	1.00 (0.60, 1.00)
	60	0.95 (0.90, 0.97)	0.96 (0.89, 0.98)	0.93 (0.87, 1.00)
	100	0.95 (0.92, 0.97)	0.96 (0.92, 0.99)	0.96 (0.92, 0.96)
0.50	20	0.95 (0.85, 1.00)	1.00 (0.90, 1.00)	0.90 (0.80, 1.00)
	60	0.95 (0.92, 0.97)	0.97 (0.93, 1.00)	0.93 (0.87, 0.97)
	100	0.94 (0.92, 0.96)	0.98 (0.96, 1.00)	0.92 (0.88, 0.96)
0.75	20	0.80 (0.70, 0.90)	1.00 (1.00, 1.00)	0.80 (0.67, 0.87)
	60	0.83 (0.73, 0.92)	1.00 (1.00, 1.00)	0.78 (0.64, 0.89)
	100	0.84 (0.75, 0.90)	1.00 (0.96, 1.00)	0.79 (0.68, 0.88)

The assignment accuracy means that if we wished to estimate the proportion of the strategies in the sample, we would estimate it correctly, except when the top-to-bottom pattern is dominant. In that case, a large portion of the top-to-bottom patterns would be classified as systematic, leading to underestimation of the number of top-to-bottom patterns in the data, as can be seen in Table 2.

**Table 2:** Median and interquartile range of the proportion of patterns classified as top-to-bottom.

		p	
n	0.25	0.5	0.75
20	0.25 (0.20, 0.30)	0.45 (0.40, 0.50)	0.60 (0.50, 0.70)
60	0.27 (0.25, 0.30)	0.47 (0.45, 0.50)	0.60 (0.48, 0.68)
100	0.27 (0.25, 0.29)	0.48 (0.45, 0.50)	0.60 (0.51, 0.66)

**Figure 2:**
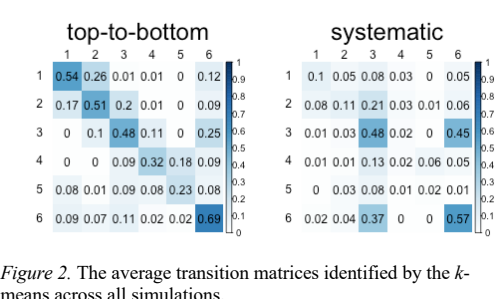
The average transition matrices identified by the k-means across all simulations.

*Stability of strategy representation.* We also inspected whether the cluster centers are stable (i.e., show relatively similar transition matrices across simulations). Table 3 shows the median and interquartile range of the pairwise Pearson's correlations between the cluster centers. Overall, the correlations are quite high, suggesting that the representations of the transition matrices remain similar across the simulations. The average cluster representations across all simulations are shown in Figure 2.

**Table 3:** Median and interquartile range of the pairwise Pearson's correlations of the cluster centers.

		Cluster label		
p	n	Systematic	Top-to-bottom	
0.25	20	0.91 (0.87, 0.94)	0.71 (0.44, 0.82)	
	60	0.97 (0.96, 0.98)	0.91 (0.87, 0.94)	
	100	0.98 (0.98, 0.99)	0.95 (0.93, 0.96)	
0.50	20	0.88 (0.84, 0.91)	0.89 (0.84, 0.92)	
	60	0.96 (0.94, 0.97)	0.96 (0.95, 0.97)	
	100	0.97 (0.96, 0.98)	0.98 (0.97, 0.98)	
0.75	20	0.77 (0.67, 0.84)	0.91 (0.86, 0.93)	
	60	0.90 (0.85, 0.93)	0.96 (0.94, 0.98)	
	100	0.93 (0.89, 0.96)	0.98 (0.96, 0.98)	

### Empirical results

Here, we present the results of the *k*-means clustering applied to the real DMM data [48] from four items. The R Script is at https://osf.io/g2yp4/. The scree plot was uninformative as it did not show a clear “elbow” pattern. Thus, we inspected the agreement between the solutions spanning from two to four clusters.

**Figure 3:**
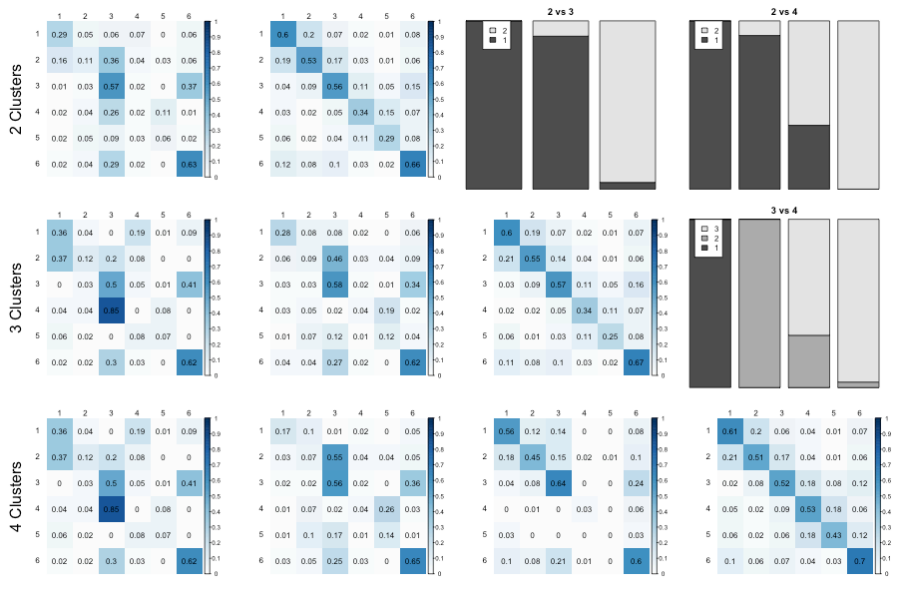
The cluster centers of the k-means solutions with two, three and four clusters, and the confusion counts of the different solutions. Each row corresponds to one solution (two, three, and four centers from top to bottom). The bars on top right of the Figure correspond to the overlap between cluster assignments. For example, panel 2 vs 3 indicates that most of the cases assigned to the first and second cluster in the three cluster solution were classified into the first cluster in the two cluster solution, whereas most of the cases classified into the third cluster with the three cluster solution were classified into the second cluster in the two cluster solution.

Figure 3 shows the average transition matrices for the two, three and four cluster solutions, and the pair-wise confusion matrices of the cluster membership. Comparing the two and three clusters solution suggests that the cluster 1 from the two clusters model is almost perfectly separated in two clusters under the three clusters model (see confusion matrix in the first row and third column). In addition, the four clusters solution finds one additional sub-cluster which is characterized by transitions between conjectures 1-3, but does not proceed further (which could be explained by the participant attempting to solve the item from top to bottom and terminating the process once the most informative feedback was found). Overall, these results suggest that the data are in line with the prediction of two general patterns – that of systematically searching for the most informative feedback, and that of attempting to solve the item in the order of conjectures as they are presented.

To check qualitatively whether the fixation sequences clustered in the groups correspond to the systematic and top-to-bottom patterns as described above, we also plot the most representative sequences for each of the clusters. We compute the “representativeness” of a sequence to a particular cluster as a Euclidian distance of the transition matrix of the sequence to that cluster center, relative to the sum of the Euclidian distances to all other clusters.

**Figure 4:**
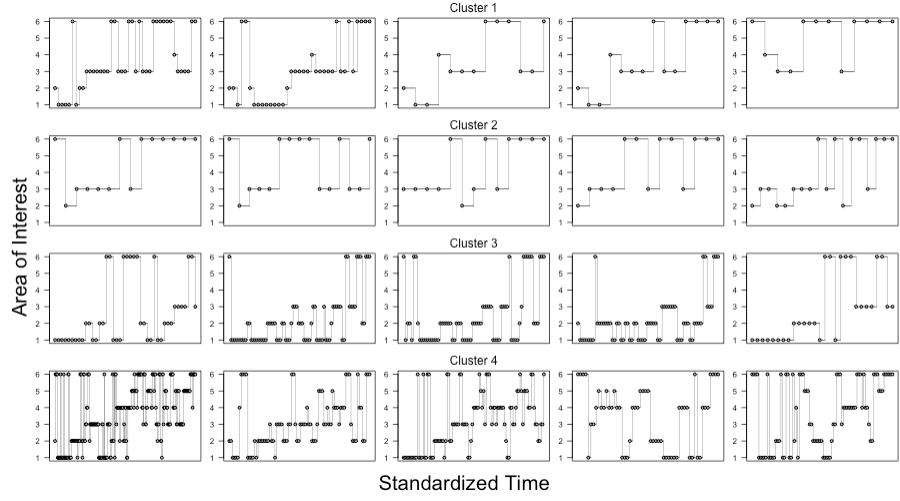
Each row shows five example scanpaths assigned to one of the clusters. Fixations to particular AOIs are shown as dots and transitions are connected with lines.

Figure 4 shows the fixation sequences, where the points show the individual fixations on particular AOIs (on the y-axis) as a function of time (time has been normalized to span between 0 and 1). Because there is a strong overlap between the cluster assignments between the 2-4 *k*-means solutions, we only show the representative fixation sequences grouped into four clusters. Clusters 1 and 2 are characterized by transitions between the third row and the response (AOIs number 3 and 6). Cluster 3 is characterized by a period of fixations on the first three rows, followed by transitions to the response. Cluster 4 is the most variable, having characteristic pattern of progression from the top to the bottom of the game with frequent transitions to the response in between. Overall, clusters 1 and 2 align with the predicted systematic patterns, whereas clusters 3 and 4 align with the top-to-bottom pattern. The distinction between the clusters 3 and 4 is that patterns in the cluster 3 usually terminate very quickly after fixating the third row (which contains sufficient information to deduce the correct solution), whereas patterns in cluster 4 do not seem to have this pattern. 

The clustering suggested that the groups also differ in terms of the number of fixations. This would be consistent with the view that the current items are relatively easy to solve when using the systematic search (i.e., focusing on the most informative feedback).

**Figure 5:**
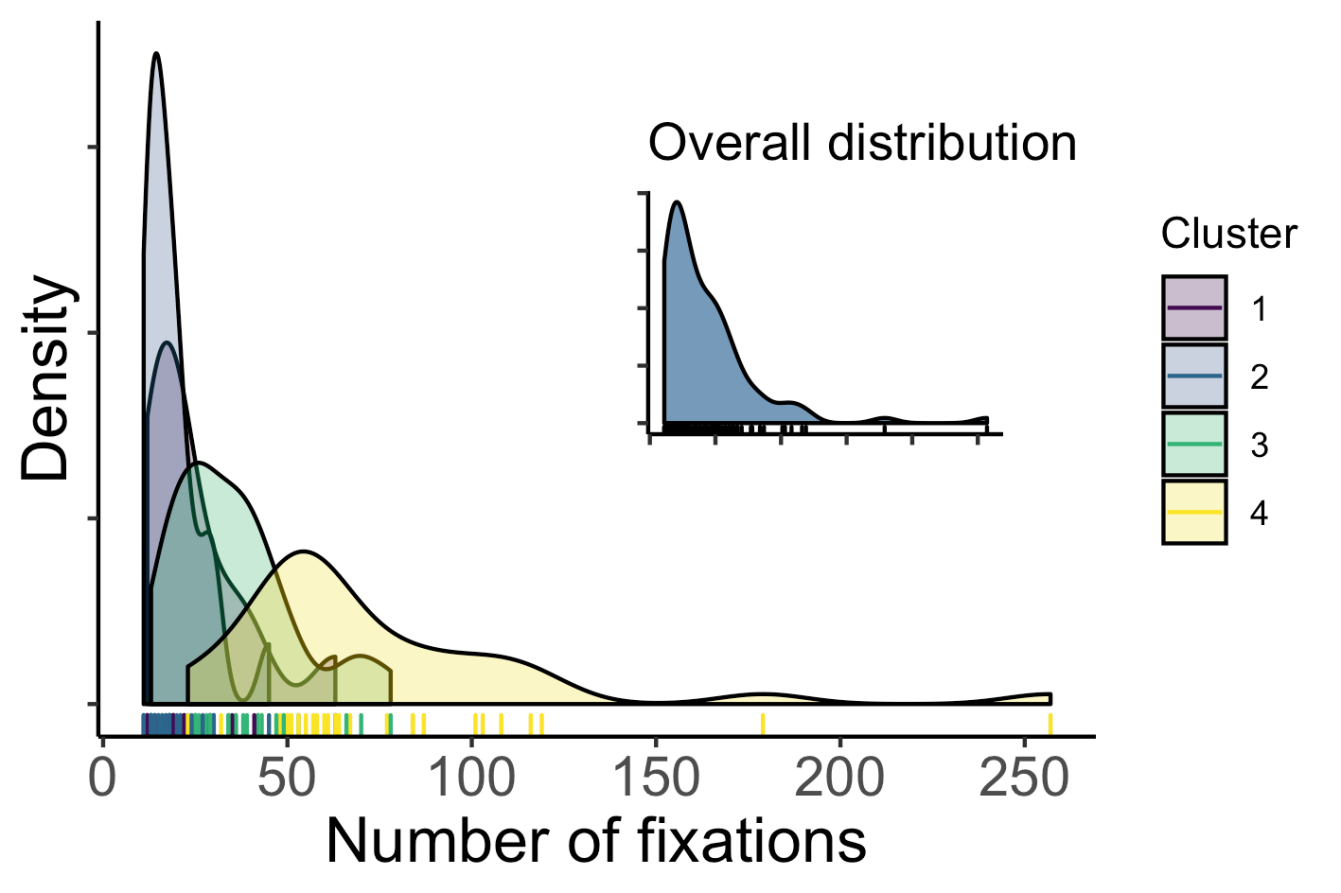
Distribution of fixations of the four clusters.

**Figure 6:**
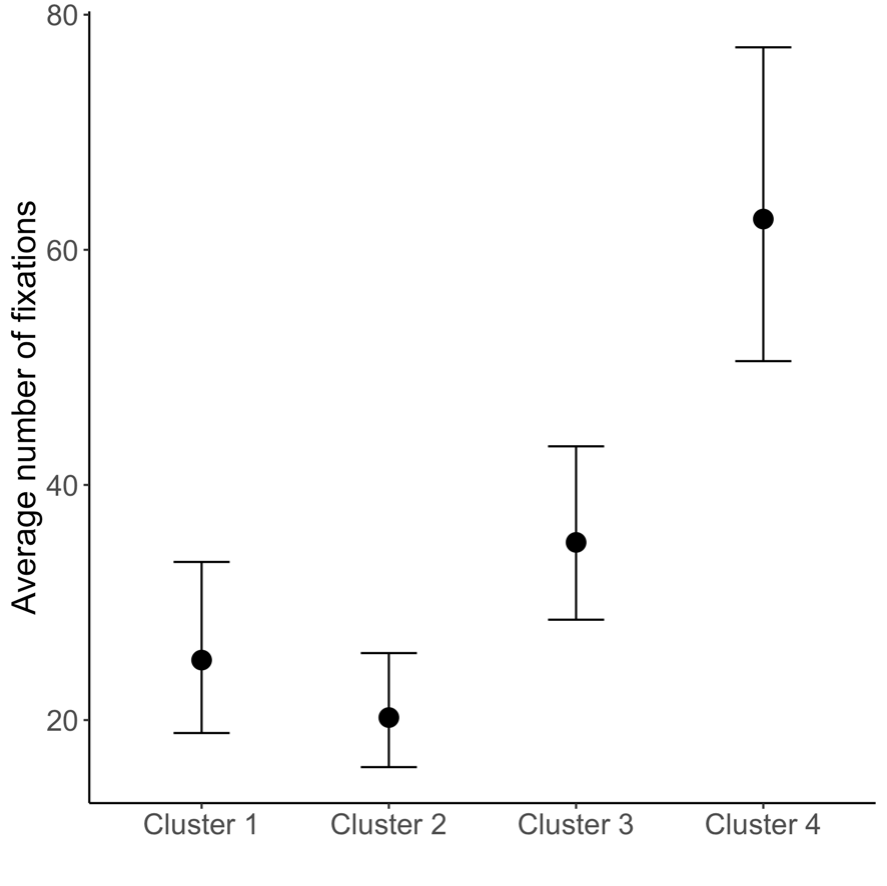
The average number of fixations of each group with 95 % credible intervals.

Figure 5 shows the distribution of the number of fixations for each cluster, as well as the marginal distribution over all data. We conducted exploratory analyses by fitting the fixation counts with multilevel negative binomial model using R package brms [57, 58] to see whether the apparent differences between the clusters are statistically supported (see https://osf.io/87ahz/). The results indicated that the cluster 4 has the highest number of fixations, the cluster 3 has the second highest, and the cluster 1 and cluster 2 are comparable, see Figure 6. However, trying to uncover the groups based on the fixation counts would be a hard task, judged by the apparent absence of multimodality of the overall distribution of fixation counts.

Lastly, we set out to investigate whether the clusters are associated with different probability of a correct answer, although the current set of items does not allow a lot of room for modelling on this part as the items are relatively easy (i.e., the percentage of correct answers is 88*.*5 %). In particular, the first three clusters had perfect or near perfect performance on these items, whereas only 68 % of the patterns in the fourth cluster resulted in correct response, see Table 4.

**Table 4:** Descriptives of the correct answers for each of the cluster.

		Cluster				
		1	2	3	4	
	n	13	23	29	31	
	# correct	13	22	29	21	
	% correct	1	0.957	1	0.677	

To sum up, we were able to cluster the real DMM data using transition matrices; in accordance with the expectations, we found two general patterns – one that is characterized by a systematic selective scanning of the most informative feedback and another characterized by a search pattern starting at the top of the item, proceeding downwards. However, the clustering results were not entirely conclusive regarding the number of clusters and it is possible that more sub patterns are hidden (i.e., one that starts as the top-to-bottom pattern and switches one the most informative feedback is processed). The patterns differ in the lengths of the sequences, and the fourth cluster seem to have different probability of correct answers. Specifically, the first two clusters have high chance of a correct answer as they appear to focus on the feedback which is sufficient to solve the item. The third cluster also has a high success rate, suggesting that it might be capturing the processes when a participant solves the item in non-systematic way (i.e., from top to bottom), but deduces the correct solution once arrived to the most informative feedback.

## Application: Progressive Matrices

In the previous example, we have shown that classifying the eye movement sequences using clustering of transition matrices is possible, even if the data are relatively sparse. To illustrate the use of clustering transition matrices in a different context, we present a reanalysis of data collected by Laurence et al. (2018). The data contain eye-tracking recordings of participants who solved Wiener-Matrizen Test 2 (WMT-2, 59, 60). The WMT-2 is structurally similar to the Raven's Progressive Matrices (RPM), as both consist of a 3 x 3 matrix containing images with varying features, where the bottom-right item is missing, and a 2 x 4 response alternatives matrix. The goal of the task is to identify which item from the response alternatives matrix belongs to the missing part of the 3 x 3 matrix, such that the varying features complete a logically consistent pattern.

Vigneau et al. [21] proposed that two distinct general strategies – constructive matching and response elimination [36]– can be employed when solving the Raven’s Progressive Matrices (RPM). The former is a systematic strategy of evaluating the matrices to deduce the only correct solution, which is then found in the response area. In contrast, response elimination is a strategy of successively considering different responses and evaluating whether they are consistent with the information given by the matrices or not. The two strategies should manifest through different eye movement patterns, as constructive matching would yield systematic transitions by rows (or columns), whereas the response elimination would show a pattern of frequent transitions from the matrix and the response area. Following the seminal work of Vigneau et al. [21], numerous studies followed up the hypothesis to replicate its findings, using mostly summary statistics from the eye-tracking data [22, 42, 43]. More recently, a different approach has been applied for describing cognitive strategies taking into account higher order dependencies in the transition patterns [23, 24]. Here, investigate this hypothesis using clustering of transition matrices.

### Laurence et al. [43] data

The data analyzed here were collected and reported previously by Laurence et al. (2018). The data are generated by 34 participants who solved 18 items (3 practice items) from Wiener-Matrizen Test 2 [WMT-2; 60]. The data contain the responses (correct/incorrect) and the processed eye-tracking data: the fixations were classified into 10 AOIs (https://osf.io/sgyk3/). The areas 1–9 correspond to the individual cells in the matrix, starting from top-left entry, and filling the matrix row-wise (e.g., 1 – top-left; 3 – top-right; 7 – bottom-left, up to 9 – bottom-right). The area 10 is the response matrix area, containing all eight options for selecting the solution. The data is organized as ordered sequences of fixations on the areas of interest for each participant and each item. If a fixation did not fall into either of the designated areas of interest, we excluded that fixation from the data, which resulted in deleting 4,338 fixations out of the total number of 91,267, leading to a 95% inclusion rate.

### Methods

Because it has been argued that in the context of Raven's Matrices, one should remove repeated fixations within one AOI, as the frequency of repeats is quite high [especially within the response matrix; 23], we use the clustering technique both on data where the repeated fixations were included (i.e., using the full data), as well as clustering data after removing the repeated fixations, essentially removing 35,880 transitions (about 44.7 %). Almost half of the excluded transitions (15,185) were based on the repeated fixations within the response alternatives matrix.

The rest of the procedure was as follows. First, each fixation sequence was converted to a transition matrix. The 34 × 18 = 612 transition matrices were reshaped into vectors and stored in a 612 × 100 data matrix. This matrix was subjected to *k*-means clustering estimating 1 to 10 clusters to inspect the scree plots.

### Results

**Figure 7:**
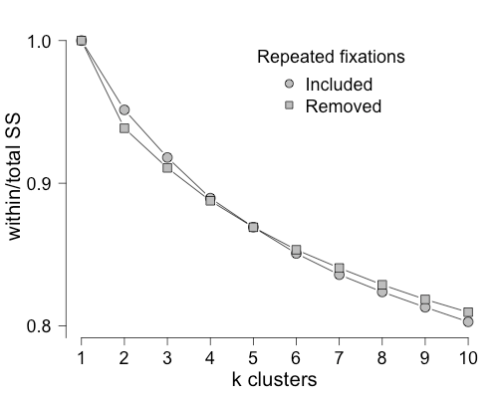
Scree plots from the k-means clustering for the data.

The scree plots from the *k*-means on data with excluded repeated fixations provided a modest support for the presence of two groups, whereas the scree plot on the full data remained inconclusive, see Figure 7. In line with previous literature [23, 24], we further discuss the results based on the *k*-means solution with two clusters; solutions with higher numbers of clusters yielded qualitatively comparable results (see https://osf.io/h3nc7/).

**Figure 8:**
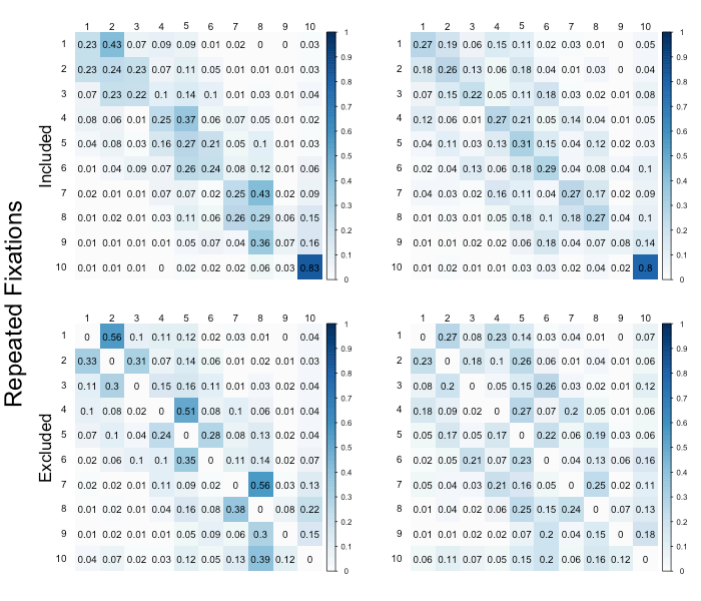
Average transition matrices of the two clusters. Left panel shows matrices of the first cluster with (top) and without (bottom) repeated fixations, right panel shows matrices of the second cluster with (top) and without (bottom) repeated fixations.

On the full data, 271 (42 %) sequences were classified into the first cluster, whereas 293 (48 %) sequences were classified into the first cluster using the data without the repeated fixations. Overall, the agreement between the two classifications was high: 516 out of the total 612 sequences (84 %) were assigned into the same cluster regardless whether the repeated fixations were excluded or not. Figure 8 shows the mean transition matrices of the two clusters. The transition matrix of the first cluster suggests a similar pattern that has been previously described by Hayes et al. [23], interpreted as the constructive matching strategy, indicating high probabilities of transitioning to left or right relative to the current fixation, which suggests a general pattern of inspecting the matrices within individual rows. However, the interpretation of the second cluster is less clear. First, the probabilities of transitioning left or right remain quite high, but there is also an increased probability to transition up or down, suggesting inspection of the matrices within columns. Second, under the expectation that the second cluster is related to the response elimination strategy, we would expect higher, and more uniformly distributed probabilities on column 10 (transition probabilities to the response area), but also in row 10 (transition probabilities from the response area). Although this is generally the case, the differences compared to the first cluster are rather small, which does not corroborate strongly that this cluster can be interpreted as the response elimination strategy.

**Figure 9:**
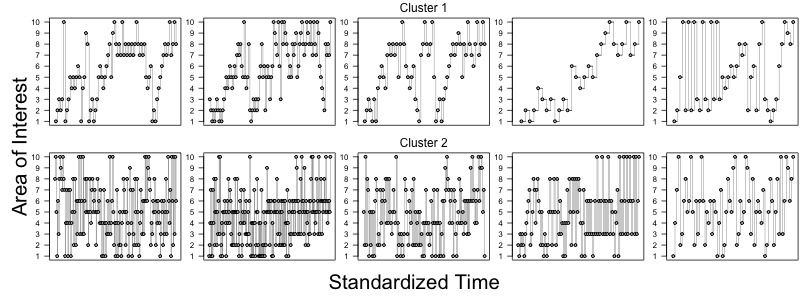
Top row shows five scanpaths that have been assigned to cluster 1, bottom row shows five scanpaths that have been assigned to cluster 2 by all three clustering methods. Repeated fixations are removed.

Figure 9 shows examples of the scanpaths that have been assigned to one or the other cluster. The first cluster is characterized by frequent transitions from left to right within rows (i.e., 1→2→3, etc.), whereas the second cluster also shows frequent transitions within columns (i.e., 1→4, 2→5, etc.).

**Figure 10:**
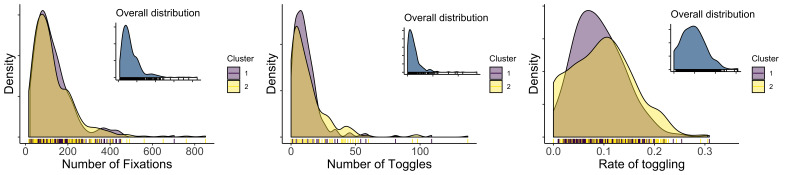
Distribution of the number of fixations (left), number of toggles (middle), and rate of toggling (right) of the two clusters.

The approach in the previous studies focusing on strategies in Progressive Matrices [21, 22, 42, 44] is to inspect, for example the number of toggles (transitions between the matrix and the alternatives), or the rate of toggling (number of toggles divided by the response time). Here, we inspected whether the two uncovered clusters differ in the length of the sequences, number of toggles, or rate of toggling (in this case defined as the number of toggles divided by the number of transitions). Figure 10 shows that the differences between the clusters are not very pronounced in either of these measures. We did not test the differences further. However, the results suggest that neither of the clusters relate to the hypothetical response elimination pattern.

Regardless of the interpretation of the clusters, we inspected their relation to performance. We fitted an exploratory multilevel logistic model using R package brms [57, 58] predicting whether the answer was correct or incorrect with a fixed and random slope for clusters, random intercept for participants and items (see https://osf.io/wvy23/). The analyses revealed that the differences between the clusters vary substantially and the average effect is not very pronounced; the second cluster performed slightly better, but the results are inconclusive. Following the focus of the original article, we fitted an exploratory model which also takes into account item types [i.e., Rule Type items, Rule Direction items, and Graphical Component Nature items; 43] and their interactions with the clusters (see https://osf.io/adt89/). Figure 12 summarizes the main results. On a descriptive level, the first cluster performs slightly better on the Rule Type items, and the second cluster performs slightly better on the Rule Direction and Graphical Component Nature items. However, these differences were very small and inconclusive given the limited sample size. We found that there was some systemacity between the cluster assignment and participants; that is, some participants were assigned consistently to one cluster over another; the number of these participants was larger than what would have been expected if participants switched between patterns randomly. Thus, we also explored the possibility that the amount of switching between the two patterns could be related to performance. However, we did not find any notable patterns. For more details, see https://osf.io/2zkj8/.

To sum up, we found two clusters in solving progressive matrices. Contrary to the results from previous literature [21, 23, 24, 43], we did not find a clear pattern that would correspond to the response elimination strategy. However, the two clusters would roughly correspond to patterns, one of which is predominantly driven by transitions within rows, whereas the other is characterized by mixtures of transitions within rows and within columns. To our knowledge, the second pattern is rarely discussed in the literature as a viable alternative to solve the matrices. It is not impossible that other, more nuanced sub-strategies remained hidden in our analysis, for example, switching between different patterns (i.e., row-wise, column-wise, and matrix-response transitions), instead of using these patterns as pure cognitive strategies.

**Figure 11:**
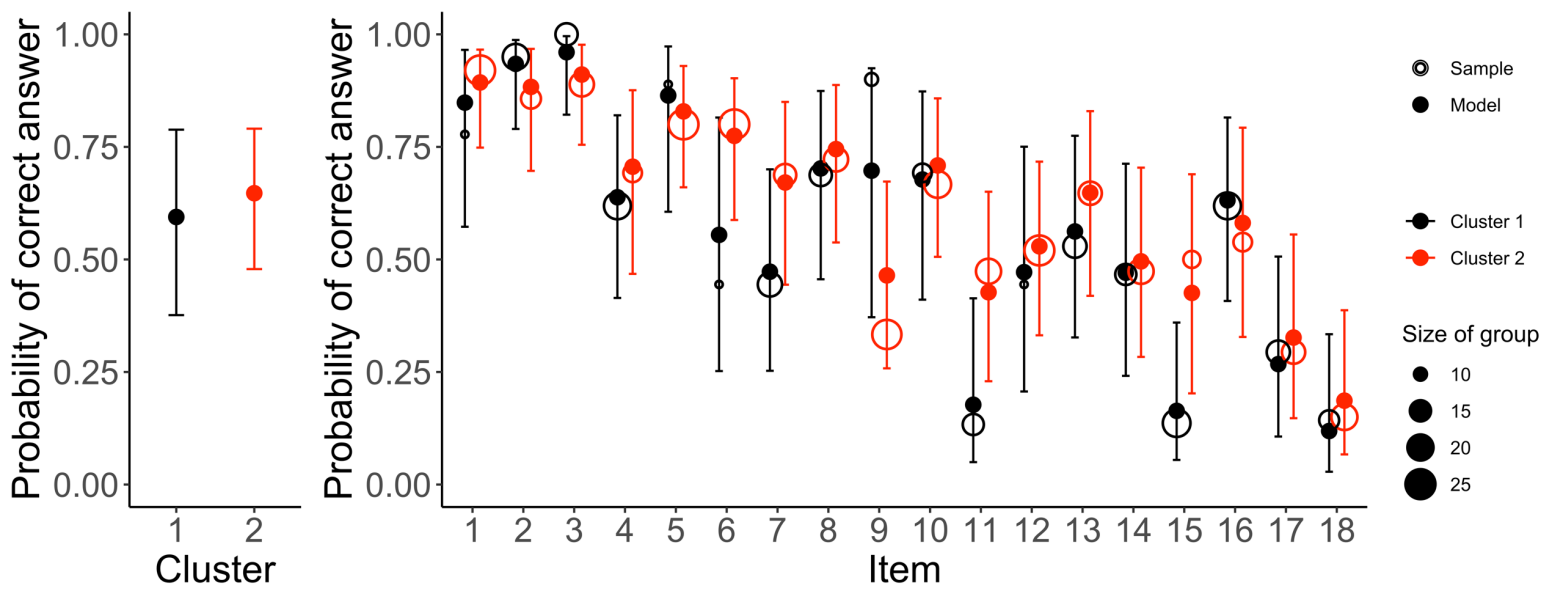
Left panel shows the marginal average probability of a correct answer of each cluster for the two clusters. Right panel shows the probability of a correct answer for each cluster and each item separately. The circles denote the observed proportion of correct answers (and the size of the circle represents the number of data points), whereas dots denote the mean of the posterior distribution. Error bars represent 95% credible intervals.

**Figure 12:**
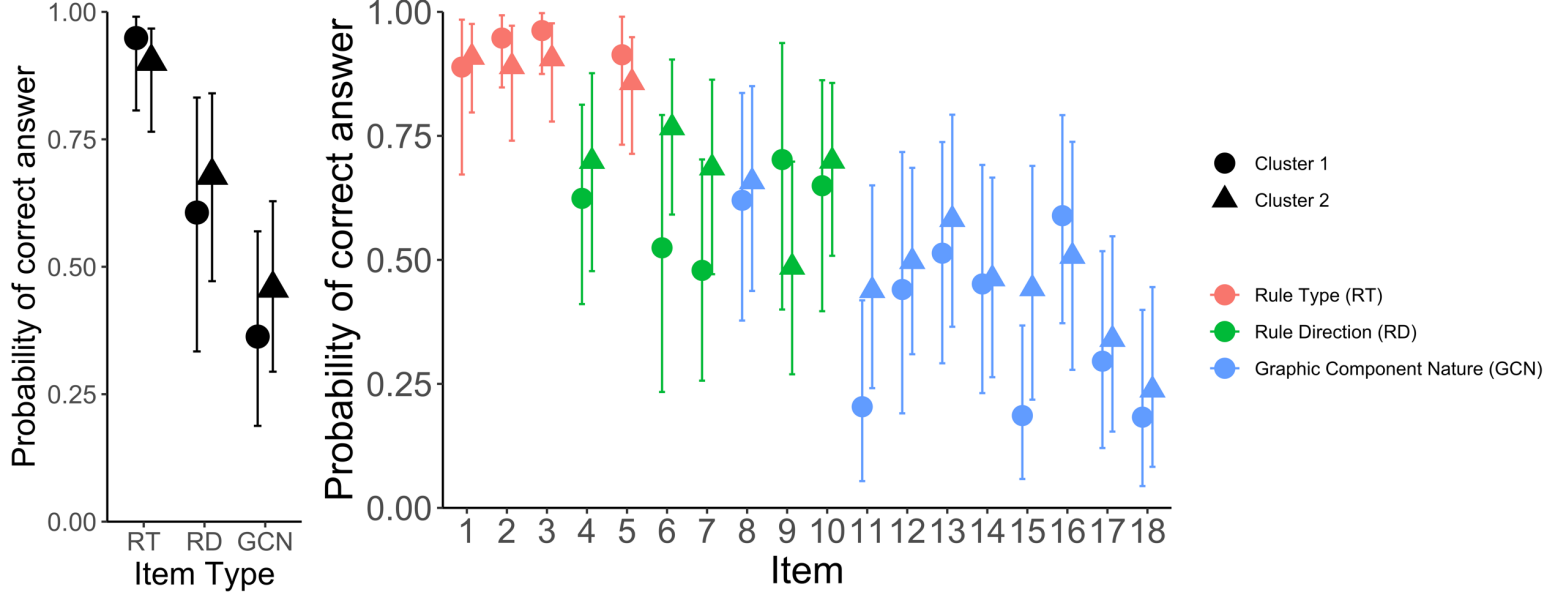
Left panel shows the interaction between the three item types and the cluster with respect to the probability of correct answer. Right panel shows the probability of a correct answer for each cluster and each item separately. Error bars represent 95% credible intervals.

## Discussion

In this article, we centralize the idea of classification scanpaths where we can assume that different strategies to solve a cognitive task could elicit different types of gaze behavior. To this end, using an unsupervised method for clustering transition matrices, we can discover groups of similar eye movement patterns without the need to assume that the groups differ on some other variable (e.g., performance in the task). This is of special interest in contexts where the groups are hypothesized and have to be inferred from the data, as well as the relationship of the group to the other variables is hypothesized and needs to be empirically tested. This problem arises frequently in the discussion of strategies in solving cognitive tasks, which we presented with two examples using the Deductive Mastermind game and Progressive Matrices task.

In the Mastermind example, we showed that we can retrieve patterns that correspond to systematic search for the most informative feedback, compared to less systematic scanning patterns guided by the order of the feedback presented. Such patterns that were predicted based on the logical reasoning analysis of the items in Gierasimczuk et al. [39]. In this example, the differences between the groups were detectable by visual inspection, which allowed us to conduct a realistic simulation study. Hence the classification should be relatively easy. From our point of view, this is a virtue of our example: showing that an automatic method arrives at the same conclusion as working through the data manually should assure us that the method is indeed valid. Furthermore, the application of the method to the real data revealed one pattern where the participant solves the item in a relatively non-systematic way, but switches to the systematic pattern once arriving at the most informative feedback, whereas another pattern suggests that the participant attempts to solve the item in a relatively non-systematic way, and does not recognize that the third row is sufficient to arrive at the conclusion.

The second application used data from Progressive Matrices items. We found a pattern corresponding to the one described in the previous literature [solving analytically the matrices by progressing through the rows, 23, 24], and one additional pattern that can be roughly described as progressing through the matrices within their columns, a pattern that has not been reported previously. Inspecting solutions with more clusters yielded qualitatively comparable results suggesting that we were unable to detect any additional patterns except these two. The patterns we found did not show differences on various summary measures (derived from eye movements, but also the performance in the task), thus, it would be hard to disentangle these patterns using supervised or semi-supervised methods, which have been predominantly used in earlier attempts to discover strategies in similar cognitive tasks [21, 22, 23, 24]. Contrary to the previous literature, we did not find a pattern that would correspond to the response elimination strategy. It is possible that the chosen representation of eye movement patterns (i.e., transition matrices) is unable to detect the response elimination pattern. Another option could be that the response elimination pattern occurs rarely as a pure strategy, but is rather emerging as a short phase during solving the items, after more systematic phases (e.g., that a person falls back on the response elimination after he or she fails to deduce the correct solution using analytic matching). If this is the case, our method could miss this pattern as it assumes that the eye-movements follow one pattern throughout solving the individual item (i.e., it is not possible to detect switches between patterns during solving the task). We believe that a comprehensive re-analysis of existing data sets [21, 22, 23, 24, 43] using a range of different methods, or a (large-scale) replication study might be appropriate to find the common ground for the findings.

Our choice of the specific representation of the eye movement data, and the methods for clustering as well as the distance metric is up for a debate. Different analytic choices could yield different results, depending on the questions and context of the analysis. We used transition matrices because the predicted strategies should differ in the transition matrices, hence, it should be possible to identify them as such. However, using transition matrices requires pre-defined areas of interest, and thus the method is limited only to applications where these areas can be defined without many arbitrary decisions. In these situations, transition matrices are simple to construct and interpret, although this should be done with caution. Some authors [23] suggested that looking only at first-order transition probabilities is too much of a simplification of the eye movements data. Further, even very different scanpaths can have similar transition matrix [12]. Thus, there is an intrinsic epistemological asymmetry – it is easier to discover qualitatively different groups of eye movement patterns than to provide evidence that some hypothesized pattern is missing (as is the case of our application on the Progressive matrices). To some extent, this asymmetry would likely occur regardless of the representation of eye movements as there will be potentially always some aspect of the data that has been left unmodelled. Individual researchers thus need to make informed decisions what representation of eye movement data to use, and if possible, commit to the analysis in advance to enable confirmatory analyses [61]. Exploratory analyses using different analytic approaches and eye movement representations can be then used to complement, expand, or challenge the confirmatory findings and their theoretical underpinnings [62] – especially if methods that build upon different assumptions lead to different results. We hope that the method we demonstrated in this article enriches the analysis toolbox for latent inverse Yarbus problem and will offer new insights, as we showed in our two examples.

The *k*-means clustering method based on minimizing squared Euclidean distances was chosen based on purely pragmatic reasons. It may be thought that the *k*-means is not the most appropriate method for clustering transition matrices, as it corresponds to the simplest form of mixture model for multivariate normal data - whereas transition matrices are essentially multivariate vectors of probability simplicia. Furthermore, the selection of the number of retained clusters with scree plots is somewhat arbitrary, and the *k*-means assumes that the groups are of equal size, leading to a bias (and potentially incorrect classification) if that is not the case. These limitations can be tackled with more advanced modeling, either by specifying a full (hidden) Markov model and use clustering techniques on them [29, 30], modeling the data as mixtures of categorical time-series where the transition matrices can be thought of as collections of multinomial variables [53], or mixture modeling of even more complex time-series models [e.g.,^63^]. Whereas either of these methods would probably do more justice to the data, we believe that simpler methods such as the *k*-means might be useful. Computing transition matrices is a simple task and the *k*-means is implemented as a basic algorithm in most of the statistical software, can be run without extensive modelling experience and knowledge, and thus is widely available to all researchers. Thus, the method we proposed can prove to be a simple alternative to assess hypotheses about qualitatively different groups of scanpaths, or explore whether the data set comprises of homogeneous eye movements patterns. Furthermore, the method is able to capture the patterns on single item basis, which we have also shown using simulations. This enables us to potentially investigate within-person variability in the cluster assignment (e.g., due to effects of learning). Further, even within the simple approach of *k*-means, there may be possible important improvements, such as using clustering based on different distance measures, different criteria for selection of the number of clusters [e.g., 64], or regularized *k*-means or *k*-means with variable selection to tackle the dimensionality of the data and identifying features important for detecting differences between the clusters [e.g., 65, 66].

While the method’s advantages perhaps facilitate its use in wide range of application, it provides only limited options for modeling the eye movement data in more flexible manner. In particular, we cannot fix certain parameters to balance over-fitting and under-fitting, nor can we take into account hierarchical structure of the data (i.e., participant and item characteristics). This limitation proved to be important in our Mastermind example, where the non-systematic, top to bottom strategy should more or less exhibit similar pattern across all items, whereas the systematic strategies should exhibit different patterns depending on the structure of the feedback. This is why we limited our example to only four items where the systematic strategy should elicit the same pattern. We could partially solve the problem by recoding AOIs on some items to conform to the same expected transition matrix, but it would not solve the problem in general. On the other hand, more flexible approaches to modeling the transition patterns would enable us to fix the strategies across items for one cluster, but let vary the strategies across items for another. Furthermore, more advanced modeling techniques could be used to identify or extend models of response behavior that assume latent states of different cognitive processes [e.g., 67, 68, 69], some of which were partially motivated by the results of eye-tracking studies on cognitive tasks [70]. However, we believe that even simple methods such as the method proposed in this article provides new ways to analyse data and derive new hypotheses, as well as think about novel directions of the eye-tracking applications.

### Contributions

Maartje E. J. Raijmakers, Ingmar Visser, and Šimon Kucharský provided the original idea of this article and wrote the manuscript. Šimon Kucharský conducted all analyses reported in this article. Martina Zaharieva provided feedback to the manuscript and ensured that the associated code is correct and reproducible. Gabriela-Olivia Truțescu collected and cleaned the Mastermind data and provided feedback to the manuscript. Paulo G. Laurence cleaned and processed data for the Progressive Matrices example and provided feedback to the manuscript and analyses.

### Ethics and Conflict of Interest

The authors declare(s) that the contents of the article are in agreement with the ethics described in http://biblio.unibe.ch/portale/elibrary/BOP/jemr/ethics.html and that there is no conflict of interest regarding the publication of this paper. 

### Acknowledgements

Šimon Kucharský was supported by the NWO (Nederlandse Organisatie voor Wetenschappelijk Onderzoek) grant no. 406.10.559. Paulo G. Laurence was supported by the FAPESP (Fundação de Amparo a Pesquisa do Estado de São Paulo) grant no. 2018/09654-7 and CAPES (Coordenação de Aperfeiçoamento de Pessoal de Nível Superior). Martina Zaharieva was funded under the Research Priority Area Yield, University of Amsterdam.

### Open practices statement

The data and analysis code are openly available at https://osf.io/wvzs9/. All analyses are exploratory and not preregistered.
